# Electrostatic control over temperature-dependent tunnelling across a single-molecule junction

**DOI:** 10.1038/ncomms11595

**Published:** 2016-05-23

**Authors:** Alvar R. Garrigues, Lejia Wang, Enrique del Barco, Christian A. Nijhuis

**Affiliations:** 1 Department of Physics, University of Central Florida, Orlando, Florida 32816, USA; 2 Department of Chemistry, National University of Singapore, 3 Science Drive 3, Singapore 117543, Singapore; 3 Centre for Advanced 2D Materials, National University of Singapore, 6 Science Drive 2, Singapore 117546, Singapore

## Abstract

Understanding how the mechanism of charge transport through molecular tunnel junctions depends on temperature is crucial to control electronic function in molecular electronic devices. With just a few systems investigated as a function of bias and temperature so far, thermal effects in molecular tunnel junctions remain poorly understood. Here we report a detailed charge transport study of an individual redox-active ferrocene-based molecule over a wide range of temperatures and applied potentials. The results show the temperature dependence of the current to vary strongly as a function of the gate voltage. Specifically, the current across the molecule exponentially increases in the Coulomb blockade regime and decreases at the charge degeneracy points, while remaining temperature-independent at resonance. Our observations can be well accounted for by a formal single-level tunnelling model where the temperature dependence relies on the thermal broadening of the Fermi distributions of the electrons in the leads.

## Abstract (web summary)

The effect of temperature on charge transport mechanisms in molecular tunnel junctions is not fully understood. Here, charge transport studies of a redox-active molecule unveil multiple mechanistic regimes which may be explained by thermal broadening of the Fermi distributions of electrons in the leads.

## Graphical abstract

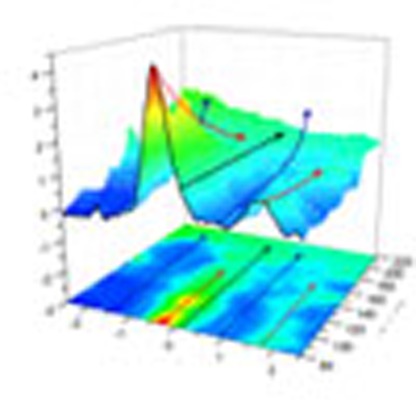

Tunnelling of electrons across molecular junctions depends on both temperature and applied bias. So far only a few systems have been reported that switch the mechanism of charge transport as a function of bias and temperature, resulting, for example, in good molecular diodes^1^,^2^,^3^,^4^ or enabling long-range charge transfer along molecular wires^5^,^6^,^7^,^8^,^9^ or biomolecules^10^,^11^,^12^,^13^. Typically, such measurements display a transition between a temperature insensitive regime, governing conduction at sufficiently low temperatures, and a thermally assisted transport regime, characterized by an exponential dependence with temperature. However, neither the origin of the thermal excitation nor the nature of the tunnelling process (coherent versus incoherent) are fully understood in solid-state junctions. For example, anomalous temperature-independent long-range (up to 16 nm) charge transport phenomena have been reported, which cannot be straightforwardly explained with current theories^6^,^12^,^13^. Hence, it is important to deepen our understanding of how temperature influences charge transport phenomena in molecular junctions and how this information can be used to improve their electronic characteristics.

Usually, temperature-independent conduction in molecular junctions is associated with coherent tunnelling involving the molecular level nearest in energy to the Fermi level of the leads (that is, the highest occupied molecular orbital (HOMO) or the lowest unoccupied molecular orbital)^5^,^14^,^15^,^16^. In this regime, the contact time of the charge carrier with the molecule is smaller than the intramolecular dephasing times. In contrast, an exponential increase of current with temperature is associated with an incoherent process, where the charge spends enough time in the molecule to allow decoherence^14^. In a wet electrochemical environment, where the current (*I*) increases with temperature (*T*) following the Arrhenius law (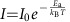, where *k*_B_ is the Boltzmann constant and *I*_0_ is a pre-exponential factor), the physical meaning of the activation energy *E*_a_ is well-understood. It relates to thermal excitations of molecular vibrational modes and reorganization of neighbouring solvent molecules, (that is, inner- and outer-sphere reorganization processes, respectively^17^). In solid-state molecular junctions, however, the absence of solvent molecules leaves the physical meaning of *E*_a_ unclear^18^. Without intermolecular interactions, thermally activated interactions are restricted to coupling of the charge carrier to vibronic states of the molecular bridge, although the associated activation energies are often too low (<0.1 eV) to explain the observations. In this scenario, the value of *E*_a_ depends also on charge image effects and the corresponding re-normalization of the molecular energy levels in the junction as a result of proximity with the metallic electrodes^19^,^20^,^21^. Thermal broadening of the Fermi occupation distribution of electrons in the leads and its overlapping with the molecular levels has been invoked as a cause of thermally activated conduction through a single-molecule tunnel junction^22^,^23^.

By far, most temperature-dependent charge transport studies have been conducted with two-terminal junctions based on self-assembled monolayers (SAMs) or single molecules contacted by conductive probes. These approaches so far only made it possible to investigate a limited range of temperatures close to room temperature and lack a gate electrode^1^,^24^,^25^,^26^,^27^,^28^,^29^,^30^. Although molecular single-electron transistors (SETs) obtained via mechanical or electromigration techniques have a gate electrode enabling full control over the energy-level alignment of the system, these junctions are normally kept at cryogenic temperatures to ensure stability^22^,^31^,^32^,^33^,^34^,^35^,^36^. It is therefore necessary to expand such temperature-dependent measurements in individual molecules by studying molecules that have been well characterized with other techniques (for example, SAM-based junctions) for comparative analysis.

Here we report electromigrated three-terminal junctions with single molecules where the conduction level is well localized within the molecule and isolated from the electrodes. We use a broad range of source-drain bias and gate voltages to study the temperature dependence of the junction across three conduction regimes: (i) the Coulomb blockade regime (where the molecular level lies outside the conduction-bias window); (ii) at the charge degeneracy point (where the level lies in between the electrostatic potential of the leads); and (iii) at resonance (where the molecular level matches the energy of one of the leads).

## Results

### Junction fabrication

**Figure 1:**
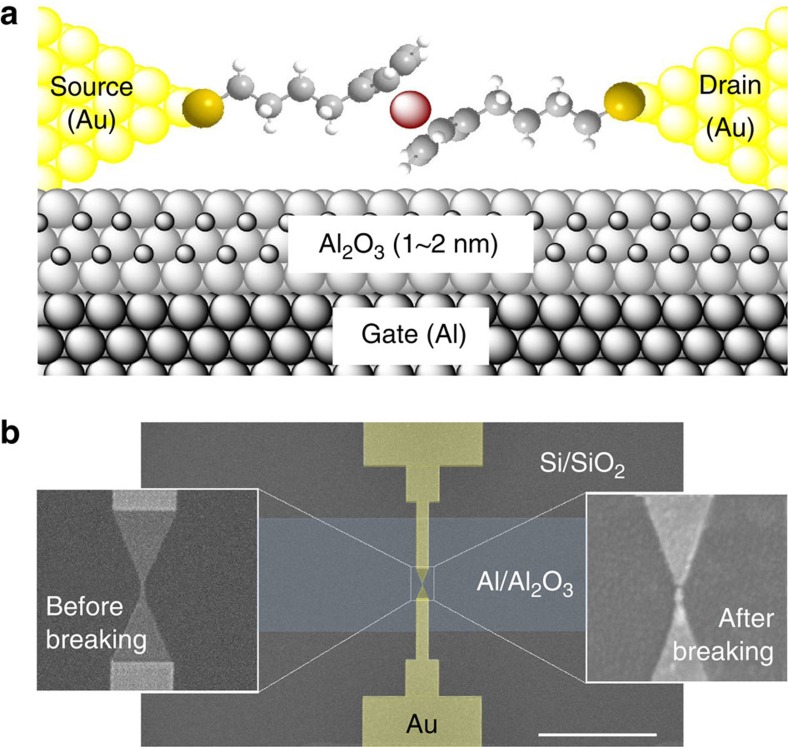
The single molecule junctions. (**a**) Schematic of a S-(CH_2_)_4_-Fc-(CH_2_)_4_-S molecule bridging the nanogap between the two bias leads of a SET. (**b**) False colour scanning electron microscopy (SEM) image of one of our SETs showing the gold nanowire on the Al/Al_2_O_3_ back gate. The scale bar is 10 μm. The insets show the thinnest part of nanowire before and after the feedback-controlled electromigration-induced breaking. The molecule completes the device by bridging the resulting nanogap during deposition from solution.

Figure 1a shows schematically the single-molecule transistor with a S-(CH_2_)_4_-Fc-(CH_2_)_4_-S (Fc=ferrocene) molecule used in this study (see Supplementary Methods). Similar molecules have been systematically studied in SAM-based junctions^1^,^2^,^3^,^37^,^38^,^39^,^40^, showing that at least three CH_2_ units between the Fc and thiolate-metal bond are needed to localize the HOMO on the Fc unit^3^. In our experiments, the molecule is connected to the source and drain electrodes via a metal-thiolate bond and the redox-active Fc unit is well separated from the leads by two -(CH_2_)_4_- tethers to avoid delocalization of the HOMO, which is centred at the Fc. Figure 1b shows an SEM image of a three-terminal SET fabricated by means of electron-beam and optical lithography^41^ (see Methods section for details).

### Charge transport measurements

**Figure 2:**
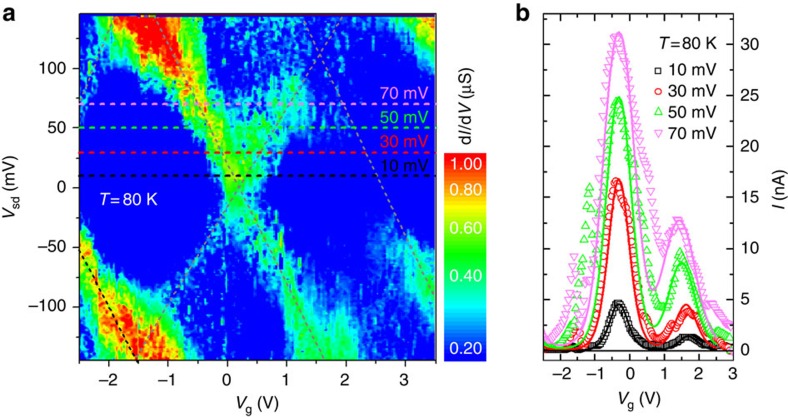
Charge transport at 80 K. (**a**) Differential conductance of a junction with S-(CH_2_)_4_-Fc-(CH_2_)_4_-S at *T*=80 K. The colour code represents the conductance (d*I/*d*V*) through the SET as a function of *V*_sd_ and *V*_g_. The grey dashed lines indicate the main resonant excitations, crossing at two visible charge degeneracy points at *V*_sd_=0 (that is, *V*_g_=0.25 and 2.5 V) and separating the Coulomb blockade areas. A third charge degeneracy point is estimated to lie around −3.5 V. (**b**) Current versus gate voltage for four different bias voltages (10, 30, 50 and 70 mV). Symbols represent experimental values and solid lines are fits to the single-level tunnelling transport model (equation 1) using the parameters given in Table 1. The colour code corresponds to the horizontal dashed lines in **a**. The molecule most likely changed its conformation within the SET electrodes between both measurements resulting in a shift in the position of the degeneracy points (new positions: *V*_g_=−0.3 and 1.7 V).

Figure 2a shows the differential conductance (d*I/*d*V*) of the molecule at 80 K as a function of source-drain *V*_sd_ (−150, +150 mV) and gate *V*_g_ (−2.5, +3.5 V) voltages. Closed diamonds characteristic of single-electron transport are clearly observed, with resonant transport excitations crossing at zero bias at two visible degeneracy points (*V*_g_=0.25 and 2.5 V), separating the Coulomb blockade areas where no current flows through the molecule (dark blue areas). Figure 2b shows the source-drain current, *I*, as a function of gate voltage for different bias voltages corresponding to the horizontal colour-coded dashed lines in Fig. 2a (the solid lines are fits to the model described below). The two consecutive charge degeneracy points start to intermix for the highest bias voltages (*V*_sd_=50 and 70 mV). The ratios of capacitive coupling *C* between Fc and the gate (g), source (s) and drain (d) electrodes can be extracted from the slopes of the resonant transport excitations (indicated by the tilted, dashed grey lines in Fig. 2a). The ratios are *C*_g_:*C*_s_:*C*_d_=1:11:13, which determine a gate coupling parameter of *g*_c_∼0.05, characteristic for a thin Al_2_O_3_ layer separating the molecule from the gate^22^. From these observations, we conclude that the Fc unit is localized in the middle of the junction (*C*_s_≈*C*_d_) and the HOMO energy level can be efficiently gated.

### Temperature-dependent transport measurements

**Figure 3:**
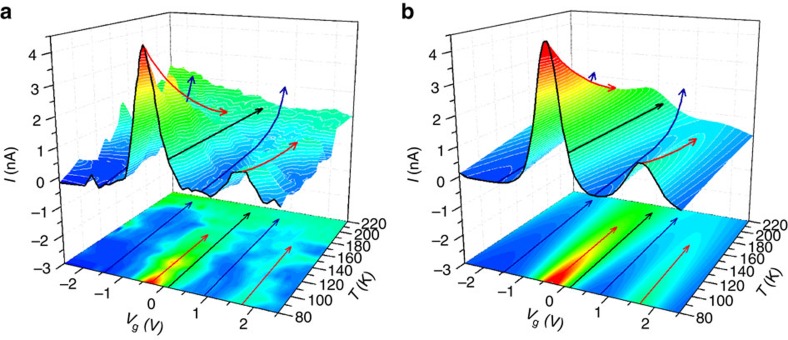
Variable temperature charge transport. (**a**) 3D plot of the evolution of the tunnel current through a S-(CH_2_)_4_-Fc-(CH_2_)_4_-S junction versus gate voltage as the temperature is increased from 80 to 220 K for *V*_sd_=10 mV. The evolution of the two charge degeneracy points (−0.3 and 1.7 V), whose magnitude decreases with increasing temperature, can be appreciated following the red arrows in both the 3D data and its 2D horizontal projection in the *V*_g_-*T* plane. Similarly, the increase with temperature in the Coulomb blockade areas can be seen by following the blue arrows. The black arrow shows the case for which the molecular level matches the electrostatic potential of one of the leads (resonance), a situation leading to almost negligible temperature dependence. (**b**) Corresponding response of the junction as calculated from the single-level tunnelling transport model in equation (1) using the parameters given in Table 1.

Figure 3a shows a 3D plot of *I* versus *V*_g_ and *T* for *V*_sd_=10 mV. The data were collected as the gate voltage was continuously swept from −2.5 to +2.5 V at each temperature, and the process repeated at different temperatures from 80 to 220 K. Similar results obtained for a second device are given in Supplementary Note 1 and Supplementary Figs 1 and 2, as well as a discussion of the reversibility of the temperature behaviour of the molecular junction in the Supplementary Note 3 and Supplementary Fig. 4. Figure 3b is the corresponding theoretical plot using the single-level model described below. From these data, we can identify three transport regimes, each of which with a distinct dependence on the temperature as indicated by the arrows: (i) the Coulomb blockade regime, with *I* increasing exponentially with increasing *T* (blue arrows); (ii) the charge degeneracy points, with *I* decreasing with *T* (red arrows); and (iii) the resonant regime (when the molecular level matches the electrostatic potential of one of the leads), with *I* constant with *T* (black arrows).

**Figure 4:**
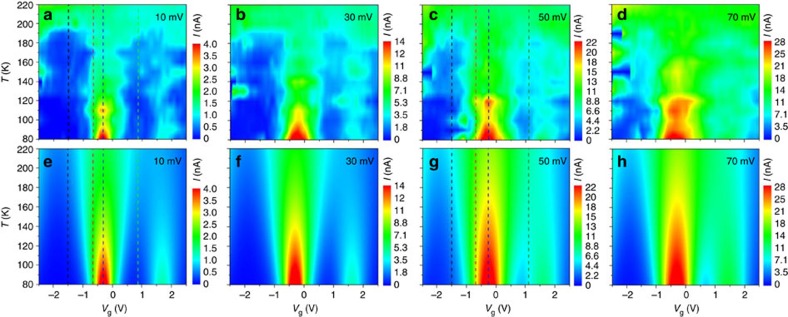
Variable temperature and source-drain voltage charge transport. Contour colour-code plots of the current as a function of the gate voltage and temperature for both experimental data (**a**–**d**) and calculations (**e**–**h**) using the single-level tunnelling transport model in equation (1) for *V*_sd_=10, 30, 50 and 70 mV. The vertical dashed lines in **a**,**c**,**e** and **d** indicate data and calculations presented in Fig. 5.

Measurements of the temperature dependence of the current were repeated at different bias values (*V*_sd_=10, 30, 50 and 70 mV). Figure 4 shows the corresponding contour plots for each bias voltage and how the transport excitations at the degeneracy points broaden as a function of bias. The magnitude of *I* exhibits an overall increase as the value of *V*_sd_ increases, as can be seen from the colour-code level bars. Still, the distinct transport regimes and their respective temperature dependencies remain present for all examined *V*_sd_ values.

### Single-level transport model

We have used a single-level tunnelling model to fit our experimental data using parameters that can be experimentally verified based on the Landauer formalism^42^,^43^. The conduction through the junction can be described as sequential tunnelling (either coherent or incoherent) from the right electrode into a single molecular level at a rate *γ*_R_, and from the molecule into the left electrode at a rate *γ*_L_, one electron at a time. The molecular level broadens to a width *γ*=*γ*_L_+*γ*_R_ as a result of its interaction with the respective electrodes. Sequential coherent tunnelling through a single-level molecular junction results in electrical current given by the expression derived by Jauho, Wingreen and Meir in 1994 (ref. 44) using a fully coherent formulation based on the Keldysh Green's function formalism:


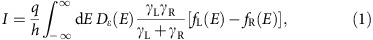


where *q* is the charge of the electron and *h* is the Planck's constant. The molecular level is described by a broadened density of states *D*_*ɛ*_ in the shape of a Lorentzian centred at the molecular level energy *ɛ*, as follows:


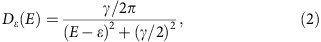


with the position of the molecular level with respect to the electrostatic potentials, *μ*_L,R_, of the transistor leads given by *ɛ*=*μ*_R_(*V*)+*ɛ*_0_+*ηqV*_sd_. Here, *η*=*V*_R_/(*V*_L_+*V*_R_) is the dimensionless division parameter giving the ratio of the voltage drop between the molecule and the right electrode with respect to the total voltage drop in the junction. In other words, *η* represents the degree of symmetry of the electrostatic potential profile across the junction, with *η=*0.5 representing a completely symmetric molecule and *η∼*0 or 1 accounting for a highly asymmetric potential drop at both sides of the molecule. In our junctions, *η=*0.5 as determined from the similar slopes of the transport excitations in Fig. 2a (that is, *C*_s_≈*C*_d_). Finally, the effect of temperature is included in equation (1) through the Fermi distributions of the electron occupation in the leads, given by:


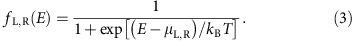


It is important to realize that for high enough temperatures (that is, 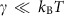), equation (1) reduces to 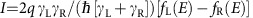, which is the exact result one would obtain using rate equations to describe incoherent tunnelling through a single level in a molecular junction^42^,^43^. Indeed, in the case that only one molecular level is involved in the conduction through the junction (which is the case in the absence of intramolecular charge relaxation), coherent and incoherent descriptions of the process become indistinguishable for all temperatures if one also considers level broadening when solving the rate equations. Note that vibrational excitations of the molecule itself would open new channels for conduction, however, the low energies associated with these excitations (<30 meV; ref. 45) allow renormalizing the molecular level by simply increasing *γ*_L,R_ in equation (2).

### Interpretation of the temperature dependencies

To explain the temperature behaviour of the current in the junction, we fitted all data to the model given by equation (1) using the parameters listed in Table 1 (solid lines in Figs 2b, 3b and 4e–h). Three molecular levels were used to account for the three degeneracy points observed within the window of electrical potentials. In essence, only two free-fitting parameters are used per molecular level to fit the results with equation (1), that is, the respective tunnelling rates *γ*_L,R_. The energy levels, *ɛ*, are independently determined from the experimental data in Fig. 2a.

The energetic difference between the main two energy levels Δ*ɛ*_1,2_ (=*ɛ*_1_−*ɛ*_2_)=160 mV (for *V*_sd_=10 mV), decreases slightly with increasing *V*_sd_ (140 meV for *V*_sd_=70 mV). This reduction in the value Δ*ɛ*_1,2_ has been observed in other molecular tunnel junctions^22^, and is associated with the static Stark effect^46^. This shift was taken into consideration in the fitting procedure. The third level, *ɛ*_3_, responsible for the degeneracy point outside the gate voltage window (*V*_*g*_∼3.5 V) is included to account for the increase in conduction for *V*_*g*_<−2 V, but its position with respect to *ɛ*_1_, that is, Δ*ɛ*_1,3_ (=*ɛ*_1_−*ɛ*_3_), was kept constant in the model as its exact value could not be measured accurately. Highly asymmetric tunnelling rates *γ*_L_≠*γ*_R_ for the main two levels had to be used to fit the results, with values similar to those reported for other molecular SETs^22^. The reason lies in the fact that these rates present two main effects on the conduction through the junction. On one hand, the sum of the right and left tunnelling rates determines the zero-temperature width of the level at each bias *(γ=γ*_L_*+γ*_R_), and therefore the highest rate is fixed by the experimental widths of the transport excitations. On the other hand, the lowest rate limits the current through the junction, and therefore is fixed by the experimentally observed value.

## Discussion

**Figure 5:**
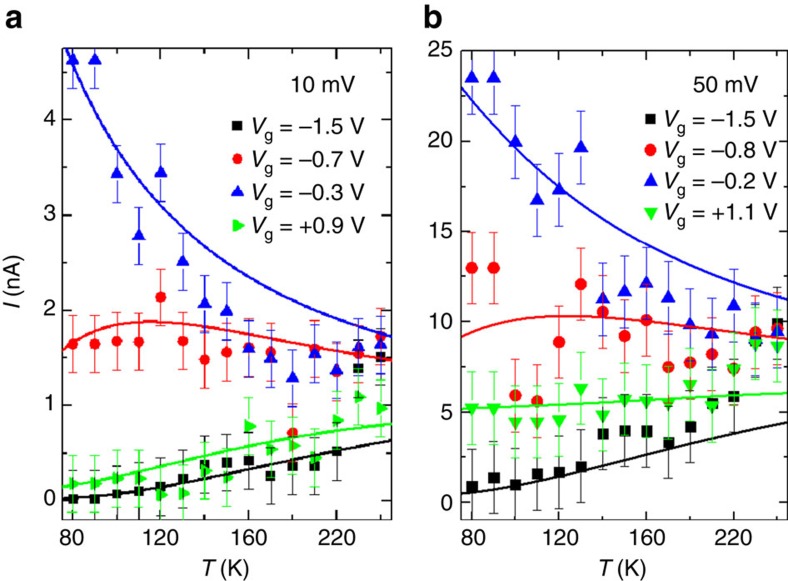
Tunnelling current as function of temperature and applied bias. (**a**) Evolution of the tunnel current with temperature at *V*_sd_=10 mV for four different gate voltages (*V*_g_=−1.5, −0.7, −0.3 and +0.9 V). (**b**) Same for *V*_sd_=50 mV (*V*_g_=−1.5, −0.8, −0.2 and +1.1 V). The solid lines in both panels represent fittings to the single-level tunnelling transport model in equation (1) with the parameters given in Table 1. The error bars represent the uncertainty in the determination of the value of the tunnelling current due to the noise of the measurements.

Figures 2, 3, 4 show the excellent agreement between the experimental data and the model, which indicates that the complex temperature dependence of the tunnelling current in all three transport regimes arises from the thermal broadening of the Fermi electronic occupation distribution in the leads. The quantitative degree of agreement between experiment and theory is easier to appreciate in Fig. 5, which displays the behaviour of *I* as a function of *T* for two bias voltages (*V*_sd_=10 and 50 mV, respectively) with the values of *V*_g_ indicated by vertical lines in Fig. 4a,c specifically selected to sample the different conduction regimes. These figures clearly show how the electrostatic potential controls the conduction regime across the molecule: (i) the junction is in the Coulomb blockage regime for *V*_g_=−1.5 (black squares) and +0.9 V (green triangles) for *V*_sd_=10 mV (Fig. 5a) and for *V*_g_=−1.5 and +1.1 V and *V*_sd_=50 mV (Fig. 5b). In this regime, *I* exponentially increases with *T* (see Supplementary Note 2 and Supplementary Fig. 3 for a discussion of a crossover into a temperature-independent transport as the temperature is further decreased in this regime); (ii) the junction is at a charge degeneracy point for *V*_g_=−0.3 V and *V*_sd_=10 mV (Fig. 5a) and *V*_g_=−0.2 V and *V*_sd_=50 mV (Fig. 5b). In this regime, *I* decreases with *T*; and, finally (iii) the junction is at resonance for *V*_g_=−0.7 V and *V*_sd_=10 mV (Fig. 5a) and *V*_g_=−0.8 V and *V*_sd_=50 mV (Fig. 5b). In this regime, *I* depends weakly on *T*.

In conclusion, our measurements reveal a complex temperature-dependent behaviour of charge transport phenomena in a ferrocene-based single-molecule junction over a wide range of bias and gate voltages. Exceptionally, we find three transport regimes in which the tunnelling current across the molecule increases, decreases, or remains constant as a function of temperature, depending on whether the junctions are tuned into the Coulomb blockade, the charge degeneracy point or the resonant transport regimes. All of our observations can be rationalized by a Landauer-type single-level model, where the complex temperature-dependent tunnelling behaviour can be explained by the broadening of the Fermi distributions accounting for the occupation energies of electrons in the transistor leads. The results described in this article represent an important step forward in explaining the temperature-dependent charge transport measurements observed in SAM-based and other single-molecule tunnelling junctions and hopefully help to improve existing models of charge transport. Currently, we are investigating junctions with asymmetrically positioned Fc moieties inside the junctions.

## Methods

### Synthesis of AcS(CH_2_)_4_Fc(CH_2_)_4_Sac

We prepared AcS(CH_2_)_4_Fc(CH_2_)_4_SAc from native ferrocene in three steps using well-established procedures (see Supplementary Methods, Supplementary Fig. 8 and refs 47 and 48). All compounds were characterized with ^1^H NMR, ^13^C NMR and ESI HRMS. Briefly, ferrocene was substituted with 4-bromobutanoyl chloride via a Friedel-Crafts acylation to yield (Br(CH_2_)_3_CO)_2_Fc (1.5 g, 45% yield). Next, this compound was reduced with borane-tert-butylamine to yield (Br(CH_2_)_4_)_2_Fc (1.2 g, 88% yield). The bromine functionality was converted to a thioacetate functionality with potassium thioacetate and the AcS(CH_2_)_4_Fc(CH_2_)_4_SAc was isolated with a yield of 95% (1.0 g).

This thioacetate derivative is stable and was stored under an atmosphere of N_2_ at −20 °C. We found that the isolation of the free dithiol was problematic because of disulfide formation. Hence our choice to isolate and use the thioacetate protected derivative, which was deprotected *in situ* for self-assembly of the junction.

### Device fabrication

For the fabrication of the SETs thin gold nanowires (15 nm thick, 50 nm wide) patterned via electron-beam lithography were deposited on top of an Al/Al_2_O_3_ back gate electrode (1–2 nm-thick oxide barrier)^41^. We used a feedback-controlled electromigration-induced breaking procedure to narrow the nanowires. Once the current dropped close to the universal single-channel conductance (*G*_0_), the current flow was stopped at which point the devices were left untouched for 2 h during which the gold atoms reorganize due to surface tension to finally create stable gaps of 1–2 nm. After the self-rupture of the wires, the chip was immersed in a 1 mM solution of AcS(CH_2_)_4_Fc(CH_2_)_4_SAc in 20 ml toluene and 5 ml methanol along with 10 mg of *n*-Bu_4_NCN (CAS 10442-39-4). Here, *n*-Bu_4_NCN in this toluene/methanol mixture deprotects the thioacetate mildly^49^,^50^. Finally, the devices were mounted in a low temperature probe and cooled down to liquid nitrogen temperatures. Out of 322 devices, 11 (∼3.5%) showed molecular transport characteristics, but only two junctions (∼0.6%) were stable enough for studies as a function of temperature (80–220 K shown in the main text, and 80–250 K shown in the Supplementary Note 1 and Supplementary Fig. 1), with molecular conformational changes as the most possible cause for device instability in the other cases. We note that charge transport measurements of molecular SETs at elevated temperatures are challenging because of the high chances for the molecule to change its conformation. We also want to remark that the yield in our experiments is similar to those in most reports of molecular single-electron transistors (see, for example, refs 22, 45, which are most closely related to this work). To ensure that our devices contained a ferrocene-based molecule, we include in the Supplementary Information details of the junctions fabrication and characterization of the nano-gaps (Supplementary Methods and Supplementary Fig. 5), detailed information on the protocols for recognition and selection of ferrocene-based junctions, including measurements with magnetic fields dependent transport (Supplementary Methods and Supplementary Fig. 6), and tests of the electrical characteristics of empty junctions as a function of temperature (Supplementary Methods and Supplementary Fig. 7).

### Transport measurements

Transport measurements were performed in DC by applying the bias voltage and measuring the current via a Keithley 6430 sub-femtoamp remote sourcemeter, while a Keithley 2400 was used to gate the transistor. The device was mounted on the sample holder of a home-made measuring probe with temperature control immersed in a liquid nitrogen dewar.

### Data availability

All relevant data are available from the authors upon request.

## Additional information

**How to cite this article:** Garrigues, A. R. *et al*. Electrostatic control over temperature-dependent tunnelling across a single-molecule junction. *Nat. Commun.* 7:11595 doi: 10.1038/ncomms11595 (2016).

## Supplementary Material

Supplementary Information Supplementary Figures 1-8, Supplementary Table 1, Supplementary Notes 1-3, Supplementary Methods and Supplementary References

## Figures and Tables

**Table 1:** The fitting parameters used to model the experimental data*.

***V***_**sd**_ **(mV)**	***ɛ***_**1**_† **(mV)**	***γ***_**1L**_ **(mV)**	***γ***_**1R**_ **(mV)**	***ɛ***_**2**_† **(mV)**	***γ***_**2L**_ **(mV)**	***γ***_**2R**_ **(mV)**	***ɛ***_**3**_† **(mV)**	***γ***_**3L,R**_ **(mV)**
10	25	1	0.120	185	3	0.040	−225	0.20
30	25	4	0.155	180	7	0.042	−225	0.24
50	25	7	0.160	170	15	0.060	−225	0.27
70	25	10	0.170	165	20	0.075	−225	0.30

^*^The model is given by equation (1).

^†^The values of the three molecular levels *ɛ*_1_, *ɛ*_2_ and *ɛ*_3_, were experimentally determined from *I* versus *V*_g_−*V*_sd_ data.
