# Supplementary material for: Electrostatic control over temperature-dependent tunnelling across a single-molecule junction
Source: Nat Commun. 2016 May 23;7:11595. doi: 10.1038/ncomms11595 (PMC4879245; doi:10.1038/ncomms11595)
Supplement: Supplementary Information — Supplementary Figures 1-8, Supplementary Table 1, Supplementary Notes 1-3, Supplementary Methods and Supplementary References [file ncomms11595-s1.pdf]

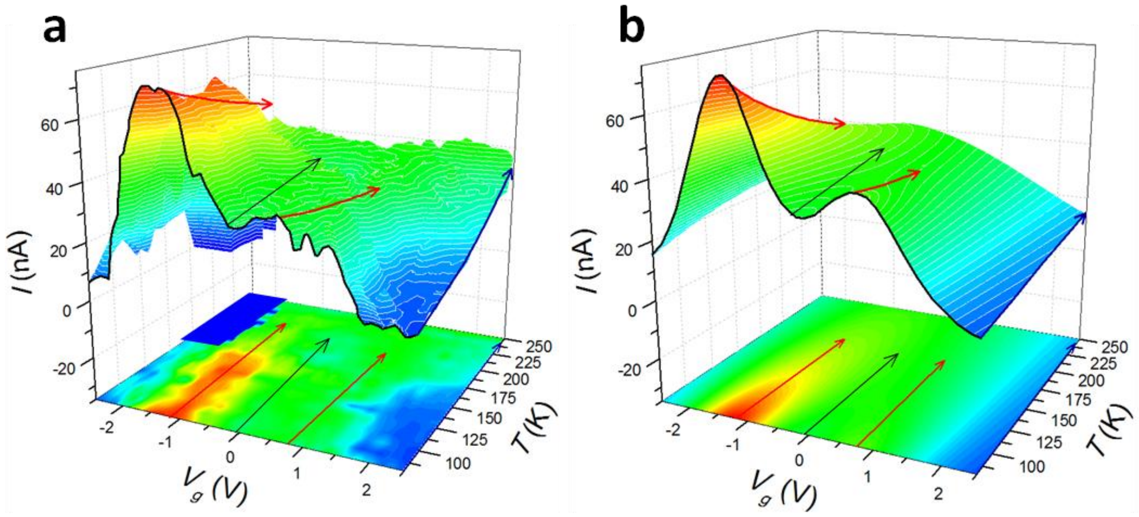

Supplementary Figure 1. a, 3D plot of the evolution of the tunnel current through a second S-$(CH_2)_4$-Fc-$(CH_2)_4$-S junction vs. gate voltage as the temperature is increased from 80 to 250 K and with an applied bias voltage of 10 mV. The evolution of the two charge points (V_g = -1.25 V and 0.8 V), whose magnitude decreases with increasing temperature are indicated with red arrows in both the 3D data and the corresponding 2D horizontal projection in the V_g-T plane. Similarly, the increase of the current in the Coulomb blockade area is indicated with the blue arrow. **b,** Corresponding response of the junction as calculated from the single-level tunneling transport model in Eqn. (1) in the main text using the parameters given in Supplementary Table 1.

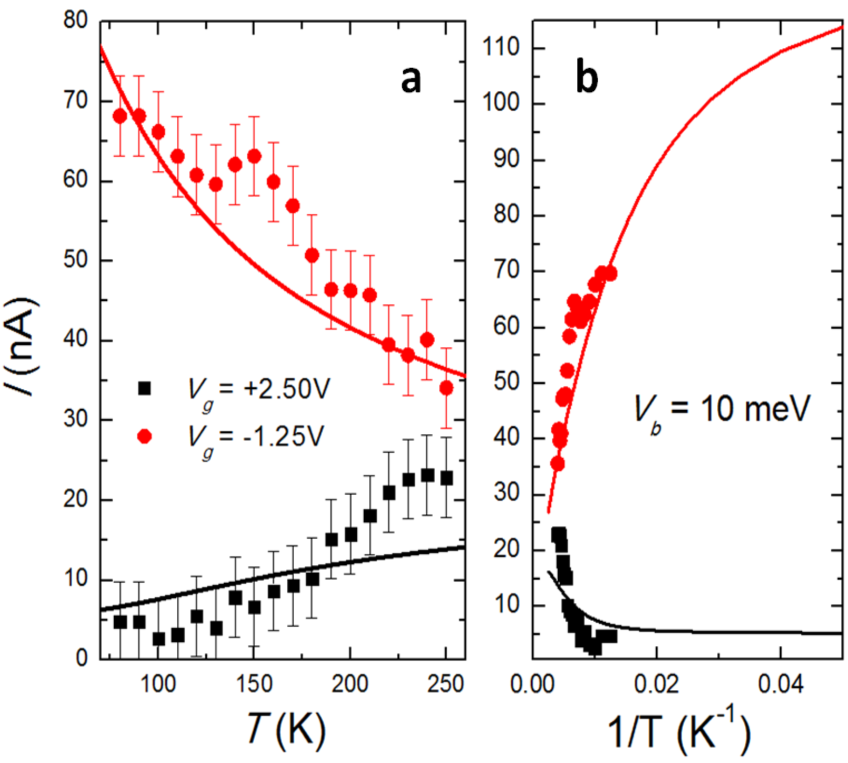

Supplementary Figure 2. a, Experimental and calculated evolution of the tunnel current with temperature at 10 mV-bias for two different gate voltages, V_g = -1.25 V (charge degeneracy point) and +2.5 V (Coulomb blockade regime). **b,** The same results shown as a function of the inverse temperature in an extended range of temperature to visualize the transition between the temperature-independent and –dependent conduction regimes, which for this molecule occurs at ~100 K. The error bars represent the uncertainty in the determination of the value of the tunneling current due to the noise of the measurements.

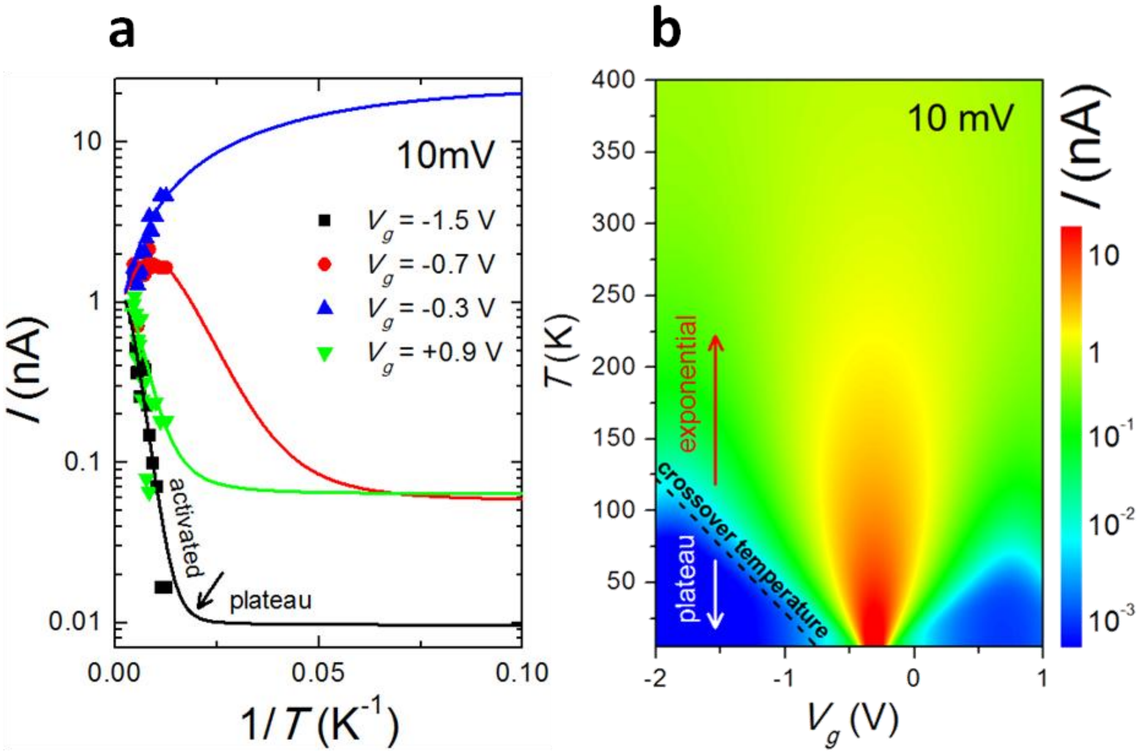

Supplementary Figure 3. a, Experimental (symbols) and calculated (lines) current versus the inverse of temperature for four different gate voltages (V_g = -1.5, -0.7, -0.3 and +0.9 V) at V_b = 10 mV. **b,** Contour color-code plot of the temperature behavior of the current (in log-scale) with respect to gate voltage for V_b = 10 mV, illustrating how the crossover between the thermally-assisted and temperature-independent regimes varies with the applied gate.

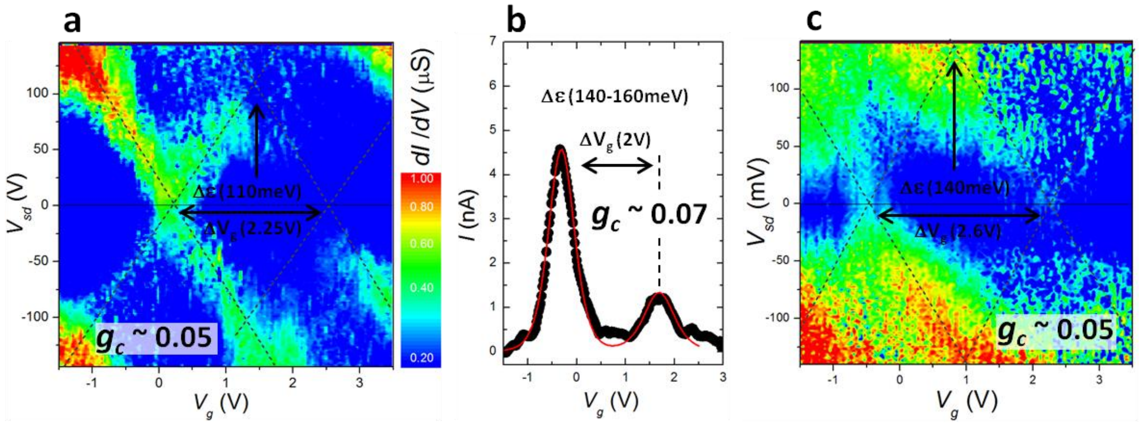

Supplementary Figure 4. a, Differential conductance of a junction with S-$(CH_2)_4$-Fc-$(CH_2)_4$-S at T = 80 K (same data as in Fig. 2a in the main text). The color code represents the conductance (dI/dV) through the SET as a function of V_{sd} and V_g. **b,** Current vs. gate voltage for V_{sd} = 10mV at T = 80K obtained after the first thermal cycling (same data than in Fig. 2b in the main text). **c,** Differential conductance of the same junction at T = 80 K after the temperature dependency study had been completed (using the same color code scale than in **a** for the conductance).

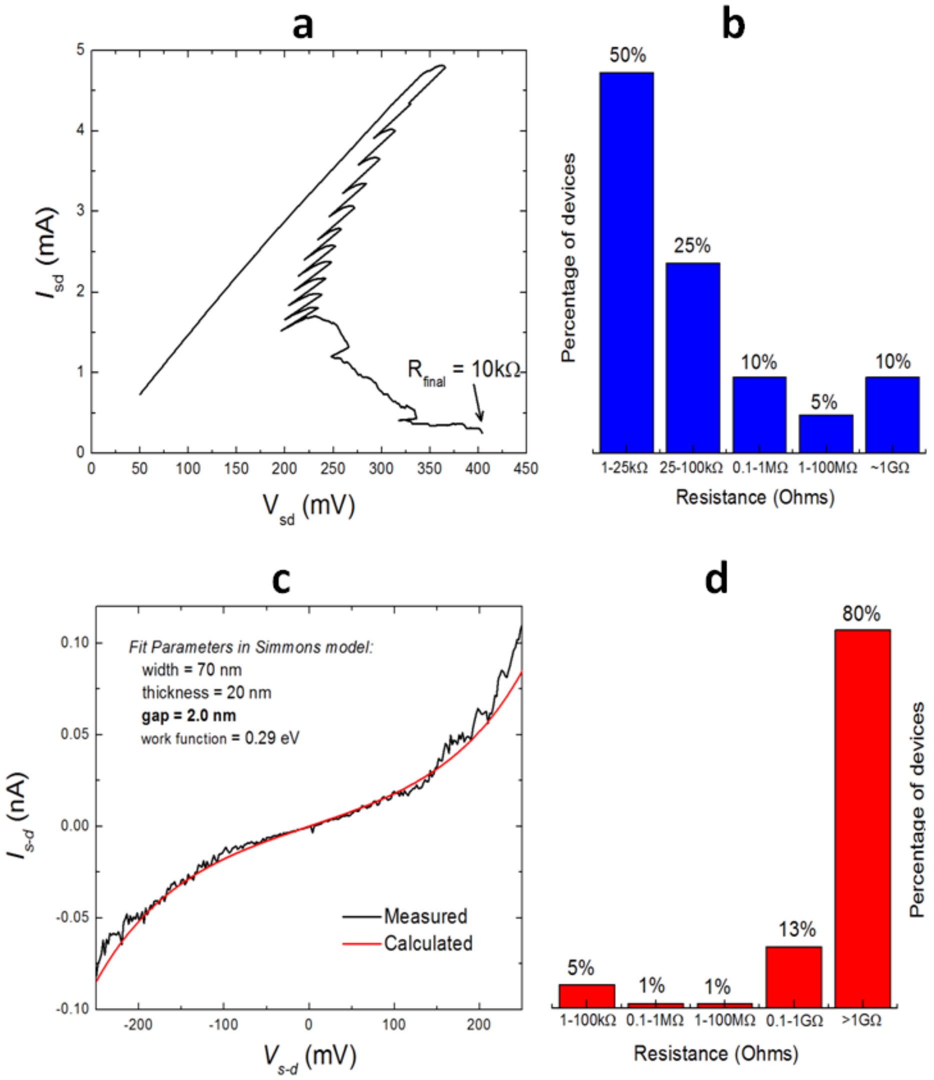

Supplementary Figure 5. a, Electron-migrating narrowing of a Au nanowire. **b,** Statistics showing the resistance of the resulting constricted wires. **c,** Tunneling current through a 2nm Au nanogap, as determined by fitting to the Simmons model. **d,** Statistics of the resulting nanogaps.

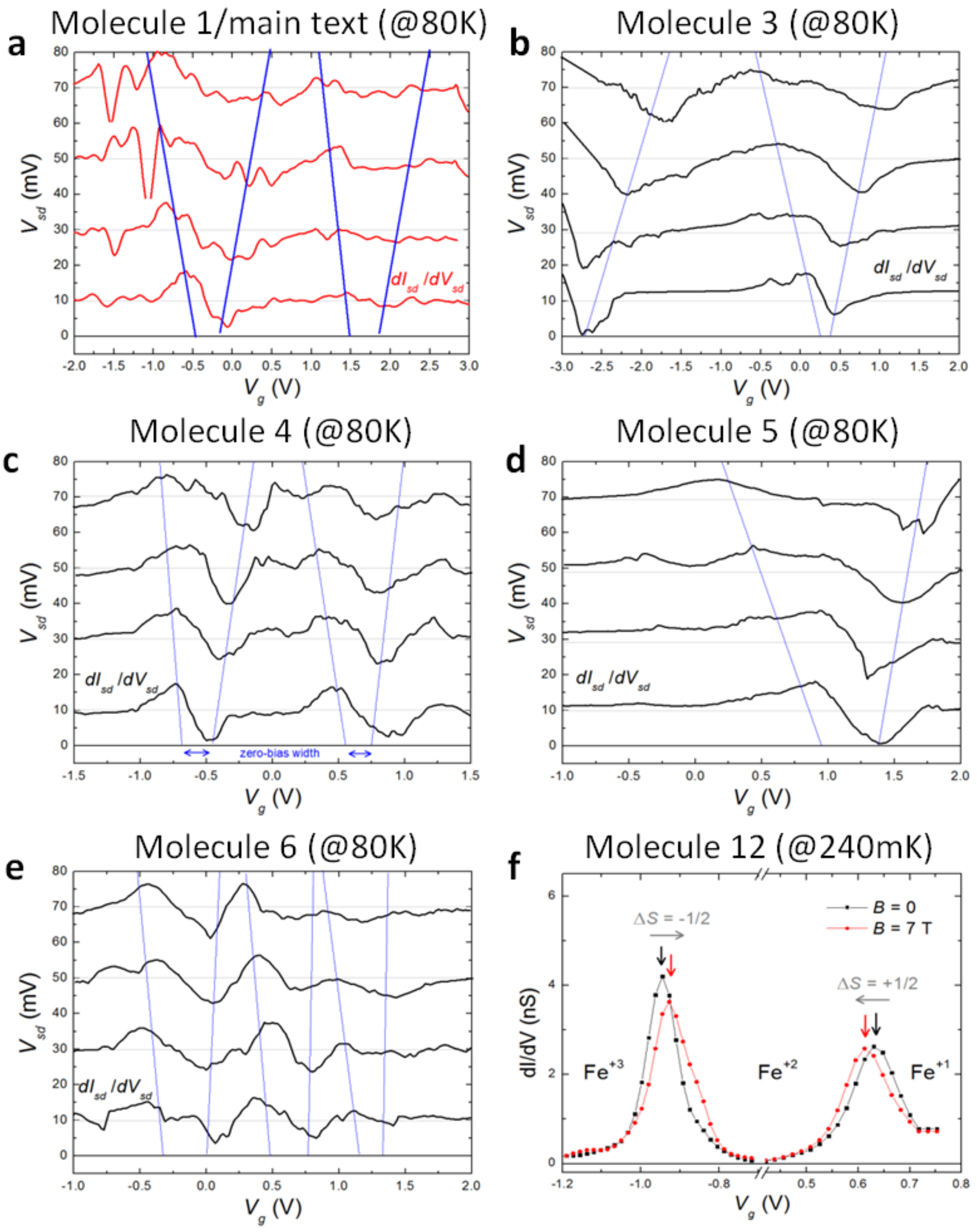

Supplementary Figure 6. a, Differential conductance as a function of gate voltage for several bias voltages (10, 30, 50 and 70mV) recorded at 80K for the molecule described in the main text of this article (molecule 1), corresponding to the derivative (dI_{sd}/dV_{sd}) of the data in Figure 2b in the main text. **b-e,** Same measurements for molecules 3 to 6. **f,** dI_{sd}/dV_{sd} vs V_g measured at T = 4K and V_{sd} = 10mV with and without a 7 Tesla magnetic field applied for another molecule.

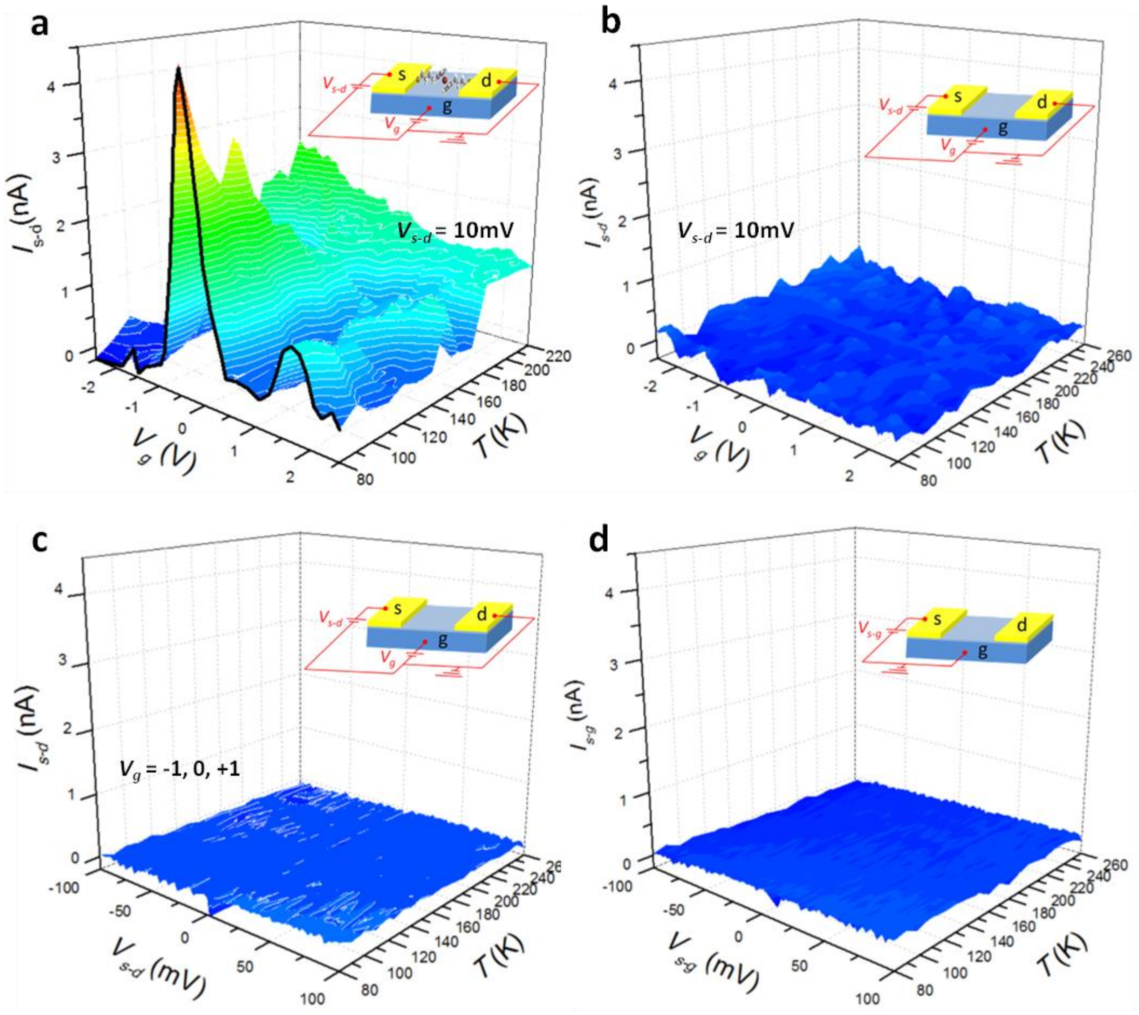

Supplementary Figure 7. a, 3D plot of the evolution of the tunnel current through a S-$(CH_2)_4$-Fc-$(CH_2)_4$-S junction vs. gate voltage as the temperature is increased from 80 to 220 K for V_{sd} = 10 mV. **b,** The same exact measurement as in (a) with an empty junction (no molecule). **c,** Tunnel current through an empty junction as a function of the bias voltage V_{sd} and temperature. The result does not change appreciably in this scale for three different gate voltages V_g = -1, 0, +1. **d,** Tunnel current between the source electrode and the gate as a function of source-gate voltage $V_{s\text{-}g}$ and temperature. The same result is obtained between the drain electrode and the gate.

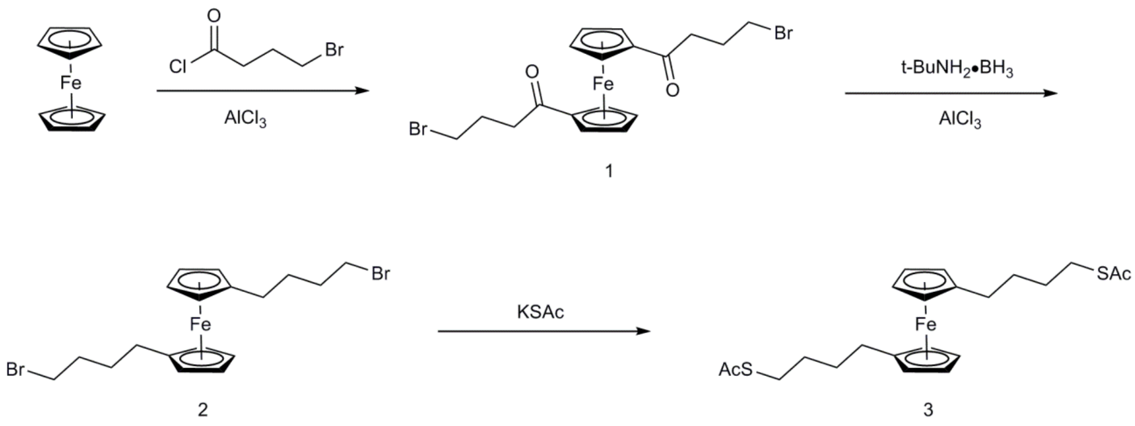

Supplementary Figure 8. Synthetic route to prepare $AcS(CH_2)_4Fc(CH_2)_4SAc$ in three steps.

Supplementary Table 1. Parameters employed in fitting the experimental data in Supplementary Figures 1 and 2 to the single-level model (Eq. 1)[a].

V_b (mV)	ε_1 (mV)	γ_{1L} (mV)	γ_{1R} (mV)	ε_2 (mV)	γ_{2L} (mV)	γ_{2R} (mV)
10	62.5	30	3	165	45	1.9

[a] Two molecular levels with energies ε_1 and ε_2 with respect to the Fermi energy of the electrodes at zero bias were determined from the experimental data.

Supplementary Note 1

SET results on second molecule

Supplementary Figure 1a shows a 3D plot of I vs. V_g as a function of temperature for V_{sd} = 10 mV in a second ferrocene-based molecule measured in this study. The data were collected as the gate voltage was continuously swept from -2.5 to +2.5 V at each temperature ranging from 80 to 250 K. Similarly to the single molecule SET described in the main text, the results allow following the behavior of the current when changing the temperature within the full range of gate voltages, enabling the analysis of the temperature dependence of the current through the junction in different transport regimes. Supplementary Fig. 1a shows the experimental data and Supplementary Fig. 1b shows the modelling results. Similar to the device described in the main text, the results show: *i*) the Coulomb blockade regime (regime 1), with I increasing exponentially with temperature (follow blue arrow at V_g = 2.5V in the S1a data and its projection in the lower V_g-T panel in Supplementary Fig. 1a); *ii*) the degeneracy points (regime 2), with I decreasing markedly with T (red arrows); and, *iii*) intermediate zones (regime 3, with just a slight variation of I with T (black arrow) when the molecular level matches the electrostatic potential of one of the leads (resonance).

The results for the second molecule were fitted to the model given by Eqn. (1) in the main text using the parameters listed in Supplementary Table 1. Supplementary Figure 1b shows a 3D plot of the calculated current through the junction. Two molecular levels were employed to account for the two degeneracy points observed within the window of electrical potentials. Again, only two free-fitting parameters were used per molecular level to fit the results with Eqn. (1), *i.e.*, the respective tunneling rates ($\gamma_{L,R}$) associated to the flow of electrons from the leads into/out of the molecule.

Here, the values of $\gamma_{L,R}$ were ~10 times larger than those used to fit the experimental data discussed in the main text; these large values of $\gamma_{L,R}$ are in agreement with the larger current magnitude (~10 times larger) and excitations widths observed in the second molecule, and we associate them to a stronger connection of the molecule with the transistor leads.

Supplementary Figures 2a and 2b display the behavior of I as a function of T for two values of V_g specifically selected to sample the coulomb blockade regime, V_g = 2.5 V (black squares), and the charge degeneracy point, V_g = -1.25 V (red circles), for V_b = 10 mV. With the same overall behaviors as those found for the molecule discussed in the main text. Specifically, with I increasing with T in the Coulomb blockade regime, and I decreasing with T at the charge degeneracy point. The transition between the temperature-dependent and temperature-independent behavior of the current in the Coulomb blockade regime can be clearly seen in Supplementary Fig. 2b (black circles) to occur at about 100 K for this molecule. The continuous lines in Supplementary Figure 2 represent the fittings to the model, in good overall agreement with the data, with the exception of the data at the highest temperatures. This is likely due to a molecular conformational change (e.g. a slight displacement of the molecule with respect to the SET leads) or a bond-fluctuation due to a change in the molecular attachment to the electrodes [1] occurring at ~175 K which shifted the charge degeneracy points ~1V towards positive gate voltages, also affecting the overall conduction through the junction for higher temperatures. To correct for this displacement, the data above ~175 K was correspondingly shifted to higher gate voltages, as can be observed in the horizontal projection of the data in Supplementary Fig. 1a, with no data in the T > 175K – Vg < -1.5V area (dark blue area).

Supplementary Note 2

Crossover between temperature dependent and independent transport in the Coulomb blockade regime

Our junctions present clear evidences of the transition between the thermally assisted and the coherent tunneling regimes in the Coulomb blockade regime (*e.g.*, V_g = -1.5 V in Fig. 5a in the main text), with the data/calculation bending as the temperature is decreased close to 80 K. Supplementary Figure 3 extends the calculations presented in the main text into lower temperatures to give a better idea of the crossover between the temperature-dependent and -independent conduction regimes. This transition depends strongly on the applied gate and bias voltages. Supplementary Figure 3a shows the case for V_b = 10 mV, where the crossover temperature in the Coulomb blockade regime (V_g = -1.5 V) lies at ~65 K, which is slightly lower than the lowest temperature used in the experiments. Supplementary Figure 3b shows a log-plot of the calculated current for V_b = 10 mV and the behavior of the crossover temperature with the applied gate voltage; this crossover linearly increases with bias.

Supplementary Note 3

Reversibility of the temperature behavior of the tunnel current through the SET

Supplementary Figure 4 shows measurements of the tunneling current through the molecular transistor described in the main text (molecule 1) obtained at different times between which the temperature of the sample had been raised to ~200K. Specifically, Supplementary Fig. 4a contains the same data shown in Fig. 2a (main text), corresponding to the preliminary characterization of the Coulomb blockade response of the molecular SET at ~80K, with the two charge degeneracy points at V_g = 0.25 and 2.5V (V_{sd} = 0), and an energy separation between the corresponding charge states of Δε ~110meV. After that preliminary characterization, the temperature was raised to ~200K, while taking some sample I_{sd}-V_g curves at V_b = 10mV at a few intermediate temperatures as a quick check of the temperature behavior of the device and to test its stability (procedure repeated with all detected molecules). After that, the temperature was decreased back to ~80K. Supplementary Fig. 4b shows the I_{sd}-V_g curve at V_b = 10mV obtained after that (same data in Fig. 2b in the main text). Only a small change in the position of the degeneracy points (now at V_g = -0.3 and 1.7V), and a change in energy difference between the two charge states (now Δε ~140meV) was observed, while the overall behavior of the molecular SET was unaltered. As discussed above, this may be associated to small conformational changes of the molecule within the transistor leads or bond-fluctuations due to alterations in the attachment of the molecule to the respective electrodes [1]. Subsequently, the temperature behavior of the device was studied while slowly increasing the temperature up to 220K, from which the central results of this work where obtained. At the conclusion of the study, the temperature was decreased again back to ~80K and a new Coulomb blockade contour plot measured (Supplementary Fig. 4c), which shows that the molecule behaved

similarly after the thermal cycling (with V_g = -0.4 and 2.2V, and Δε ~140meV), demonstrating the reversibility of its temperature behavior.

Supplementary Methods

Electromigration-breaking of nanowires and device statistics

For the fabrication of the nano-gap SETs we follow a recipe that we have refined over recent years by which gaps separating the source and drain transistor leads of the order of 1-2nm are achieved in high yields (>80%) at room temperature, which is similar to that reported by van der Zant and collaborators [2]. For this, a feedback-controlled electromigration breaking procedure (Supplementary Fig. 5a) is employed first to narrow the wires down until obtaining resistances ranging from 1 to 100kΩ in most of the wires, 75% (*i.e.*, the wire is not completely broken). Supplementary Figure 5b shows the statistics corresponding to the wires prepared for the studies presented in this article (with a sample of over 300 nanowires).

After the narrowing of the nanowires, the chips are left untouched for 1-2 hours, during which time the reorganization of the gold surface leads to the eventual rupture of the wires, generating gaps of 1-2nm in most of the wires. Supplementary Figures 5c and 5d present the characteristic tunneling current of the nano-gapped wires and the statistics on the batch used for this study, respectively. In the case of this study, 93% of the resulting wires present resistances within the vicinity of 1GΩ, indicative of 1-2nm gaps. In the original report on this procedure by van der Zant and collaborators the authors state that no evidence of formation of gold nanoparticles in the gaps was found out of 300 junctions prepared with this recipe [2].

After the gaps have been formed, a solution with the molecules under study is used to bridge the nanogaps during a 1-hour deposition process in with the solution is put in direct contact with the chip of SETs. After that, the chip is inserted into the low-temperature probe and brought to base temperature, where it is tested for variations in

the gaps' resistances (decrease) indicative of the presence of a molecule bridging the gap separating the source and drain electrodes. As mentioned in the main text, out of 322 devices, 11 (~3.5%) showed molecular transport characteristics. Note that this procedure prevents having to break the Au nanowires at low temperature, for which the molecules had to be deposited in advance. Allowing the room-temperature gold surface reorganization to complete the nanowire rupture helps in obtaining small gaps with high mechanical stability and much higher yields (~90%) than low-T breaking (with past yields limited to 50% in our group). In addition, since the rupture is performed at room temperature, the molecules can be deposited after the gap has been formed, minimizing substantially (almost eliminating) the possibility of formations of metallic islands that can be mistaken by molecules or thermal degradation of the molecule during electron migration. It is for this important reason that signatures of Coulomb blockade with charge states separated by 100meV or more are taken as signatures of molecular presence, as discussed below.

Identification of single-molecule SETs for this study

As a test routine after deposition of molecules in the nano-gapped SETs, the chip is cooled down to T = 80K and the tunnel current I_{sd} is tested at each transistor by applying a small bias voltage (V_{sd} = 10mV). A decrease of an order of magnitude or more in the tunneling current through a transistor with respect to its value before the deposition of molecules is taken as an indication of an incidence in the nano-gap.

For those transistors, the differential conductance is monitored as a function of the gate voltage for four different source-drain voltages (*i.e.*, V_{sd} = 10, 30, 50 and 50mV). Figure S6a-e shows examples of five out of the eleven molecules recognized during the studies

performed at 80K, including the molecule discussed in the main text (molecule 1), which is displayed in Supplementary Fig. 6a. As clearly observed in Supplementary Figures 6a-e, charge transport excitations are observed at different gate voltages, whose width (separation between peaks and dips in the dI_{sd}/dV_{sd}) increases as the bias voltage (V_{sd}) is increased (follow blue lines in Supplementary Figs. 6a-e). The linear dependence of the transport excitations with the bias voltage is indicative of the characteristic response of a molecular SET, with charge degeneracy points separating different molecular charge states, *i.e.*, areas with suppression of tunneling current by the Coulomb blockade effect. Identification of charge states separated by $\Delta\varepsilon$ = 100meV or more (bias voltage values for which adjacent blue lines would meet), together with the fact that the gaps are formed at room temperature in the absence of molecules, is taken as proof of a molecular presence in these eleven SET junctions. Note that strong renormalization effects are known to lower significantly (one order of magnitude) the energy separation between molecular charge states as a result of the presence of the metallic electrodes, which act as electrical mirrors. Forty nine other junctions showing gate and bias dependencies but not meeting the criteria above (*i.e.*, $\Delta\varepsilon$ < 100meV, non-linear dependence or low-resistance in the junction before the deposition of molecules) were disregarded for further study.

It is known that the variable electrostatic environment between the electrodes in the SETs makes molecules in general (and ferrocene in particular) to display different charge states after deposition [3]. Indeed, the charge state of the molecule can be tested by monitoring the relative shift in gate voltage of the charge degeneracy points under the action of an applied magnetic field, $\Delta V_c = -2g\mu_B C_T/C_g \Delta S$, where g is the electron Lande factor, μ_B is the Bohr magneton, C_T is the total capacitance of the junction, C_g is the gate capacitance, and ΔS is the spin change [4]. To check on this, a chip of forty

SETs prepared according to this protocol was mounted in a low-T probe and inserted in a dewar with a superconducting magnet and brought down to sub-milli Kelvin temperatures for studies in the presence of a magnetic field. Supplementary Fig. 6f shows the measured tunnel current through one of the chip's junction at 240mK (black data), which clearly displays two charge degeneracy points at V_g = -0.95 and +0.63 V. Note that the signal is substantially less noisy than that from measurements at 80K (molecular junctions are also more stable at low temperature). Upon application of a magnetic field of 7 Tesla, the peaks move towards different directions in gate voltage, with the first/second peak shifting to higher/lower voltages, respectively. The corresponding analysis determines a change of spin ΔS = -1/2 for the first degeneracy point, while crossing the second degeneracy point involves a positive change of spin ΔS = +1/2. The observed spin transitions agree well with the two transport degeneracies separating the following charge states in the ferrocene, Fe^{3+} (S = 1/2) $\rightarrow$ Fe^{2+} (S = 0) $\rightarrow$ Fe^{1+} (S = 1/2), and would be difficult to reconcile with a non-molecular origin. This provides further support to the association made in this work between the transport response of the transistors and the ferrocene-based molecules used to construct them.

Three-terminal SET circuit characterization and its temperature behavior

Once the characteristic molecular signatures, as those shown in Supplementary Figs. 6a-e, are identified the corresponding molecular junctions were subjected to measurements at a few temperatures between 80 and ~200 K to check the thermal stability of the junctions. As mentioned in the main text, only two molecules survived the first change in temperature (molecule 1 in main text, molecule 2 in this document),

and remained stable for a detailed study within the 80-220K (1) and 80-260K (2) *T*-ranges.

It is important nonetheless to assure that the observed temperature behavior is originated by the molecule in the junction and not by device artifacts. To check this end, the behaviors of several empty junctions (*i.e.*, SETs from the same fabrication batch but without molecules deposited) were examined upon changes in temperature. The results of one empty junction are shown in Supplementary Figure 7 (similar results were observed in the other junctions examined). For comparison, Supplementary Fig. 7a shows the contour plot of the tunnel current I_{sd} as a function of gate voltage and temperature for a bias voltage V_{sd} = 10mV (same data as in Fig. 3a in the main text). It is very illustrative to compare Supplementary Fig. 7a with 7b, since they correspond to the same exact measurement in the molecular junction (Supplementary Fig. 7a) and in the empty junction (Supplementary Fig. 7b). At all gates and temperatures (in the case of the empty junction up to 260K) the current through the empty junction remains negligible (I_{sd} < 0.1nA in all examined empty junctions) when compared to the current through the molecular device (almost two orders of magnitude larger at the charge degeneracy point, and more at higher bias voltages). Note that although the current magnitude in a molecular junction depends greatly on its coupling to the electrodes (see, *e.g.*, the much larger current amplitude in molecule 2 in this document), it is always expected to be substantially larger than that flowing across an empty gap. But, most importantly, the absence of prominent features in the temperature and gate voltage responses of the empty junctions provides the distinct behavior observed in the molecular junctions a strong proof of their molecular origin. Supplementary Figure 7c shows the evolution of I_{sd} vs. V_{sd} with temperature in an empty junction (the corresponding tunneling curve and its fit to the Simmons model for *T* = 80K was shown

in Supplementary Fig. 4c). Again, no appreciable changes are observed within the range of temperatures measured.

Finally, the devices are always checked for leaks between the source/drain transistor leads and the gate electrode. The isolation between them is achieved by a thin (1-2nm) Al_2O_3 layer which is fragile and sensitive to drastic electrostatic changes. These barriers are routinely characterized in our laboratories any time a new fabrication batch of SETs is obtained. Voltages above 4.5-5 volts are necessary to break the barrier, and the resulting leaks are easily identifiable during the SET measurements (sometimes the rupture occurs while measuring due to an electrostatic discharge). However, it is important to verify that the small tunneling current between source/drain leads and the gate is not compromised when varying temperature. To evaluate this, the tunnel current between the source and gate electrodes in several empty junctions has been measured within the same temperature range described above and the results in one of the devices displayed in Supplementary Fig. 7d (identical results were found in the other devices examined). Basically, the tunneling current is again negligible (<0.1nA) when compared to the current through the molecular junctions reported in this article and does not display any appreciable change with increasing temperature.

Synthesis detail

The following chemicals were purchased from Sigma-Aldrich and used without further purification: 4-bromobutanoyl chloride, borane-tert-butylamine complex, ferrocene, anhydrous aluminum chloride and potassium ethanethioate. Solvents for chemical synthesis were freshly distilled prior to use: dichloromethane (DCM) was distilled from calcium chloride and tetrahydrofuran (THF) was distilled from

sodium/benzophenone. Deionized water (18.2 MΩ cm) was generated from a water purifier (Purelab Option). All moisture sensitive reactions were performed under a N_2-atmosphere. Thin layer chromatography (TLC) glass plates coated with 0.25 mm thick layer of silica gel 60 and fluorescent indication UV_{254} (Macherey-Nagel) were used to monitor the progress of the reactions. The products were purified by column chromatography over silica gel (pore size 60 Å, 230-400 mesh particle size, 40-63 μm particle size, Sigma-Aldrich). ^{1}H and ^{13}C NMR spectra were recorded on a Bruker Avance 300 MHz (AV300) spectrometer using chloroform-d as a solvent. Electrospray ionization (ESI) high resolution mass spectra were recorded on a Finnigan LCQ mass spectrometer.

Compound 1

We followed a acylation procedure described in the literature [5] to synthesize ω-bromo aliphatic-1,1′-diacylferrocenes. In a 100 ml Schlenk flask, ferrocene (1.3 g, 7 mmol) and anhydrous $AlCl_3$ (2.8 g, 21 mmol) were dissolved in anhydrous DCM (30 mL), next a solution of 4-bromobutanoyl chloride (2 mL, 17 mmol) in anhydrous DCM (25 mL) was added to the reaction mixture dropwise at room temperature. The reaction mixture was stirred for 3 h at room temperature under a nitrogen atmosphere. After addition of deionized water (30 mL), the reaction mixture was stirred for an additional 10 min. The dark red colored organic layer was separated from the blue colored aqueous layer. The aqueous layer was extracted three times with DCM (25 mL) and the combined organic layers were washed with saturated sodium chloride, dried over sodium sulfate, filtered, and concentrated using rotary evaporation. The crude product was purified by column chromatography (hexane/DCM = 1:3) to yield the product (1,5 g, 45% yield) as a dark red oil.

^{1}H NMR ($CDCl_3$, 300 MHz) δ 4.83 (s, 4H), 4.54 (s, 4H), 3.58 (t, 4H, J=6.0 Hz), 2.88 (t, 4H, J=6.6 Hz), 2.26 (m, 4H); ^{13}C NMR ($CDCl_3$, 75 MHz) δ 202.1, 80.1, 73.6, 70.6, 37.5, 33.8, 26.6; ESI HRMS *m/z* calcd for $C_{18}H_{21}FeBr_2O_2$ 482.9253, found 482.9249 (M^++H).

Compound 2

The reduction was performed according to a literature [6] reported procedure. Borane-tert-butylamine complex (1.56 g, 18 mmol) in anhydrous DCM (50 mL) was added at 0 °C to a suspension of $AlCl_3$ (1.2 g, 9 mmol) in anhydrous DCM (50 mL), the resulting mixture was allowed to stir at 0 °C for 1h until a clear solution was obtained. A solution of compound **1** (1.45 g, 3 mmol) in anhydrous DCM (25 mL) was added dropwise. The reaction mixture was stirred at 0 °C for 2 h and then hydrolyzed with deionized water (30 mL). The aqueous layer was extracted three times with DCM (25 mL) and the combined organic layers were washed with 0.1 M HCl, water and saturated sodium chloride, dried over sodium sulfate, filtered, and concentrated using rotary evaporation. The crude product was purified by column chromatography (hexane/DCM = 10:1) to provide product (1.2 g, 88% yield) as a yellow oil.

^{1}H NMR ($CDCl_3$, 300 MHz) δ 4.07 (s, 8H), 3.41 (t, 4H, J=6.6 Hz), 2.29 (t, 4H, J=6.9 Hz), 1.88 (t, 4H, J=6.9 Hz), 1.65 (m, 4H); ^{13}C NMR ($CDCl_3$, 75 MHz) δ 89.3, 69.3, 68.5, 33.8, 32.5, 29.6, 28.5; ESI HRMS *m/z* calcd for $C_{18}H_{24}FeBr_2$ 453.9589, found 453.9596 (M^+).

Compound 3

In a 100 ml Schlenk flask, potassium thioacetate (0.72g, 6.25 mmol) and compound 2 (1.15 g, 2.5mmol) were dissolved in anhydrous THF (50 mL). The solution was refluxed for 2 hour, and then allowed to cool to room temperature overnight. The

resulting mixture was poured into water and extracted with ethyl acetate. The combined organic layers were washed with saturated sodium chloride, dried over sodium sulfate, filtered, and concentrated using rotary evaporation. The crude product was purified by column chromatography (hexane/DCM = 3:1) to provide product (1 g, 95% yield) as a yellow oil.

^{1}H NMR ($CDCl_3$, 300 MHz) δ 4.04 (s, 8H), 2.87 (t, 4H, J=6.9 Hz), 2.32 (s, 6H), 2.27 (t, 4H, J=7.2 Hz), 1.58 (m, 8H); ^{13}C NMR ($CDCl_3$, 75 MHz) δ 195.9, 89.4, 69.2, 68.3, 30.6, 30.3, 29.3, 28.9, 28.8; ESI HRMS *m/z* calcd for $C_{22}H_{31}FeO_2S_2$ 447.1109, found 447.1107 (M^++H).

Supplementary References

[1] Tao, N. J. Electron transport in molecular junctions. *Nature Nanotech*. **1,** 173–181 (2006).

[2] O'Neill, K., Osorio, E. A. & van der Zant, H. S. J. Self-breaking in planar few-atom Au constrictions for nanometer-spaced electrodes. *Appl. Phys. Lett*. **90,** 133109 (2007).

[3] de Leon, N. P., Liang, W., Gu, Q. & Park, H. Vibrational excitation in single-molecule transistors: deviation from the simple Franck-Condon prediction. *Nano Lett*. **8,** 2963–2967 (2008).

[4] Hansen, W. *et al.* Zeeman bifurcation of quantum-dot spectra. *Phys. Rev. Lett*. **62,** 2168–2171 (1989).

[5] Vulugundam, G., Kumar, K., Kondaiah, P. & Bhattacharya, S. Efficacious redox-responsive gene delivery in serum by ferrocenylated monomeric and dimeric cationic cholesterols. *Org. Biomol. Chem.* **13,** 4310-4320 (2015).

[6] Gharib, B. & Hirsch, A. Synthesis and characterization of new ferrocene-containing ionic liquids. *Eur. J. Org. Chem.* **19,** 4123-4136 (2014).
